# The use of brain-specific biomarkers in urine for prediction of neurological outcome and extent of tissue damage following stroke

**DOI:** 10.1038/s41598-025-28115-1

**Published:** 2025-12-03

**Authors:** Franziska Lieschke, Daniel T. Marggrander, Maximilian Rauch, Jan Hendrik Schaefer, Ferdinand O. Bohmann, Christian Foerch, Christian Grefkes, Konstantin Kohlhase

**Affiliations:** 1 Department of Neurology, Goethe University Frankfurt, University Hospital, Theodor-Stern-Kai 7, 60596 Frankfurt Am Main, Germany; 2 Department of Anaesthesiology, Intensive Care and Pain Therapy, Sana Hospital Offenbach, Offenbach Am Main, Germany; 3 Institute for Neuroradiology, Goethe University Frankfurt, University Hospital, Frankfurt Am Main, Germany; 4 Department of Neurology, RKH Klinikum Ludwigsburg, Ludwigsburg, Germany

**Keywords:** Acute ischemic stroke, Intracerebral hemorrhage, Biomarker, Brain injury, Functional outcome, Stroke, Biomarkers

## Abstract

Serum biomarkers of neuronal and glial damage gained significant attention in recent years for their diagnostic and prognostic value in acute stroke. In this study, we explored the potential of spot urine testing as a practical, non-invasive alternative. Using ultrasensitive single-molecule arrays, we assessed levels of glial fibrillary acidic protein (GFAP), neurofilament light chain (NfL), ubiquitin carboxy-terminal hydrolase L1 (UCH-L1), and total tau (t-tau) in urine and blood samples from hospitalized patients with acute ischemic stroke (AIS) and intracerebral hemorrhage (ICH) within 96 h after symptom onset. The results were correlated with mortality rates, tissue damage extent and modified Rankin Scale (mRS) scores at discharge and after three months. Significant variables were included into an multivariate regression model with confounder correction (NIHSS at admission, age at onset and serum creatinine). As a result, absolute concentrations of urine GFAP, NfL and t-tau were significantly higher in patients with in-hospital death or death at 3 months follow-up compared to patients reaching a mRS ≤ 3. Using multivariate regressions models, urine GFAP demonstrated the strongest predictive value across all biomarkers for in-hospital functional outcome, which was superior to the respective GFAP serum concentrations, whereas NfL in serum was the only significantly predictive biomarker for 3 months functional outcome. For radiological outcomes, urine GFAP concentrations were closely linked with ischemic lesion size (ρ = 0.42, p = 0.01). In conclusion, the measurement of neuronal and glial biomarkers in urine is feasible and may enhance the prediction of functional and imaging outcomes.

**Supplementary Information:**

The online version contains supplementary material available at 10.1038/s41598-025-28115-1.

## Introduction

The glial and neuronal cell death due to intracerebral hemorrhage (ICH) or acute ischemic stroke (AIS) leads to the release of neuronal and glial biomarkers into blood^1,2^. As such, glial fibrillary acidic protein (GFAP), neurofilament light chain (NfL), ubiquitin carboxy-terminal hydrolase L1 (UCH-L1), and tau levels in particular increase in serum following acute brain damage^3–7^. Previous studies have already shown a positive correlation of these biomarkers in blood with the functional outcome and the extent of tissue damage and the feasibility (for GFAP when performed in the early phase) to even differentiate between etiologies (ICH vs. AIS)^6,8–10^. While the measurement of biomarkers in blood has widely been evaluated for this particular purpose over the last decade, there are no reliable data about the use of urine as a non-invasive alternative. In a previous study, we demonstrated that GFAP, NfL, UCH-L1, and total tau (t-tau) could be non-invasively detected in urine from healthy volunteers and patients with acute neuronal and glial damage, providing a diagonstic value in differentiating patients with acute brain damage from healthy subjects^11^. To date, it has not been investigated whether the measurement of these biomarkers in urine is also suitable for predicting mortality and functional outcome or its association with the extent of tissue damage. In this regard, the advantage of a measurement in urine is its non-invasiveness, which can be particularily helpful in structurally weaker regions. In addition, urine as an ultrafiltrate of the blood with a possible accumulation of proteins could have a different, perhaps even superior diagnostic or predictive value to a measurement in serum. The aim of this study was therefore to evaluate the correlation and predictive value of GFAP, NfL, UCH-L1 and t-tau levels in urine with the neurological outcome and the imaging based tissue damage. Furthermore, we aimed to investigate whether a distinction can be made between the respective etiologies (ICH vs. AIS).

## Methods

### Study design

This prospective monocentric study was performed between October 2019 and January 2021 at the Department of Neurology of the University Hospital Frankfurt, Germany.

Inclusion criteria were as follows: 1. confirmation of AIS or ICH by cerebral magnetic resonance imaging or computed tomography; 2. age > 18 years; and 3. blood and urine collection within 96 h after symptom onset or “last seen well”. Exclusion criteria were: 1. transient ischemic attack, ischemic stroke or ICH within the last 3 months; 2. brain tumor; 3. traumatic brain injury within the last 3 months; and 4. lack of informed consent from the patient, legal representative, or relatives.

### Clinical baseline and outcome parameters

Clinical parameters were captured as follows: diagnosis of AIS or ICH according to International Statistical Classification of Diseases and Related Health Problems 10th revision (2021), age, sex, National Institutes of Health Stroke Scale (NIHSS) score at admission and discharge, serum and urine creatinine, stroke etiology, individual pre-existing conditions and risk factors and the pre-stroke modified Rankin Scale (pre-mRS). For patients with AIS, the mode of recanalization (if performed) was registered (i.e. intravenouse thrombolysis or mechanical thrombectomy). The main outcome measures were the in-hospital mortality, the mRS at discharge and, if available, at 3 months following the index event. The extent of tissue damage (lesion volume) was detected in the follow-up cerebral imaging at 24 h (or if follow-up scans were unavailable in the initial imaging) and evaluated by an expierenced neuroradiologist by manual volumetry on a radiologic workstation (Centricity RIS-i 7 Viewer; GE Healthcare, Chicago, USA) using axial diffusion-weighted MR images (b = 1000 mm/s^2^, slice thickness 5 mm,) or axial computed tomographic images (slice thickness 5 mm), respectively. For patients who underwent mechanical thrombectomy, the reperfusion result was also recorded using the thrombolysis in cerebral infarction (TICI) score.

### Blood and urine sampling and measurements

Serum samples were drawn by venous puncture, while the concomitant urine samples were obtained as midstream spot urine or, if this was not possible, from the puncture chamber of a transurethral catheter and collected in a urine tube. The time point of collection within 96 h after symptom onset or “last seen well” was recorded. The 96-h time frame was determined based on previously reported kinetics of serum GFAP, aiming to measure the estimated serum peak of biomarkers during the early subacute phase^12,13^. After obtaining serum and urine samples, the tubes were centrifuged and the supernatant was stored in Eppendorf tubes at − 80 °C; sample collection and freezing was carried out within 4 h after collection. The Simoa^®^ Human Neurology 4-Plex A and B (N4PA, N4PB) assay (a digital immunoassay for the quantitative determination of NfL, t-tau, GFAP and UCH-L1 in human serum, plasma and cerebrospinal fluid) was used to analyze all samples. Incorrect measurements, such as saturated samples, were excluded from the following analysis. Values below the limit of detection were considered to be 0.0 pg/ml and included in the analysis. This was the case for 5 patients in the serum UCH-L1 measurements and for one patient in the urine GFAP measurement. A sensitivity analysis excluding these patients can be found in the supplemental material. Because urine collection was performed as spot urine, dilution correction was calculated using a biomarker–creatinine ratio in addition to the absolute urine concentration (biomarker/creatinine in urine [mg/dl]/[mg/dl])^11,14^.

### Statistical analysis

Statistical analysis was performed using GraphPad Prism Version 10.2.2, GNU R Version 4.1.1 as well as IBM SPSS^®^ (IBM Corp. Released 2021. IBM SPSS Statistics for Macintosh, Version 29.0. Armonk, NY: IBM Corp). Ordinal and non-normal continuous data is depicted as median with 25th and 75th percentiles (Q1–Q3), normally distributed continuous data is depicted as mean ± standard error of the mean (SEM). Frequencies of nominal data are depicted as percentages. Depending on the normal distribution and level of measurement, baseline variables and biomarker levels were compared between two outcome groups using the t test, the Mann–Whitney U test, the χ^2^ test, or if more than to groups were compared using the Kruskal–Wallis test. A p-value < 0.05 indicated statistical significance.

A multivariate ordinal regression model was applied to predict the mRS at discharge and at 3 months. Candidate covariates were identified through Spearman correlation and univariate regression analyses of serum and urine biomarkers. Variables with a p-value < 0.05 were considered for inclusion in the multivariate analysis. Each biomarker was tested in a separate model as the primary variable of interest, with adjustment for NIHSS on admission, age, and serum creatinine. Model fit of the multivariate regression models were evaluated using the Chi-square (χ^2^) statistic from the likelihood ratio test, with higher χ^2^ values indicating a greater contribution to model fit, allowing direct comparison of explanatory power across biomarkers. To control for multiple testing, p-values were adjusted using the Benjamini–Hochberg false discovery rate (FDR) procedure and reported as q-values. The same analytical strategy was applied to infarct size and hemorrhage volume (ml), using linear regression models. In addition, receiver operating characteristic (ROC) analysis was performed to determine cutoff values of serum and urine biomarkers for in-hospital mortality and 3-month mortality. For each ROC analysis, the area under the curve (AUC), sensitivity (Sn), and specificity (Sp) were reported.

### Standard protocol approvals, registrations, and patient consents

The study protocol was approved by the institutional review board of the Goethe-University Frankfurt (236/05). All procedures involving human participants were conducted in accordance with the ethical standards of the institutional review board and with the 1964 Declaration of Helsinki and its later amendments. Written informed consent was obtained from all patients or their authorized representatives before the samples were included in the study.

## Results

### Study population

**Fig. 1:**
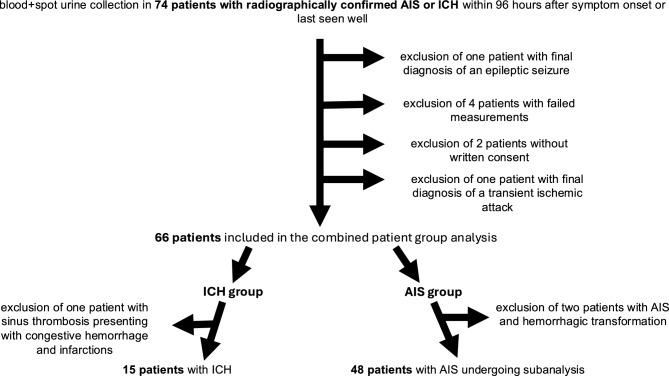
Study flow chart. *AIS* acute ischemic stroke, *ICH* intracerebral hemorrhage.

Blood and urine samples were collected from 74 patients. One patient was excluded who was initially mistakenly treated as a stroke, but the final diagnosis was an epileptic seizure with Todd’s paresis. In 4 patients, the measurements failed and no values (neither from the urine nor the blood) could be obtained. In two patients written informed consent was missing. In one patient, the final diagnosis was a transient ischemic attack, leaving 66 patients to the combined patient analysis. Two patients with AIS presented with parenchymal hematoma type PH2 according to the Heidelberg bleeding classification^15^. In one patient, the infarctions were caused by a sinus thrombosis. This patient also presented with congestion hemorrhages. These three patients were excluded from the analysis according to the stroke types due to uncertain group assignment (ICH vs. AIS, Fig. 1). From 6 patients no data on the mRS at 3 months was available (lost to follow-up rate: 8.96%). Overall, 10 patients (15.2%) of the combined patient group analysis died within the first 7 days (defined as in-hospital mortality), and 5 more (7.5%) between discharge and the follow-up at 3 months.

### Characteristics according to the respective stroke subtype (AIS vs. ICH)

Our cohort consisted of 48 patients with AIS (2 patients were excluded from this subgroup analysis due to parenchymal hematoma) and 15 patients with ICH. The median age of the patients suffering an ICH was significantly lower compared to patients with AIS (62 years [53–71 years] in the ICH group vs. 77 years [66–83 years] in the AIS group, p = 0.02). The AIS group showed a higher average initial serum creatinine level, while the ICH group tended to be more severely affected with a higher NIHSS at admission and at discharge, although these differences were not statistically significant.

The median infarct size was 36 ml (2–104 ml), the median ICH volume was 128 ml (38–294). We found a trend towards a worse functional outcome measured by mRS at discharge in patients with ICH (median mRS at discharge 5 (4–5) vs. 4 (2– 5) in the AIS group, p = 0.1) as well as in mortality, however this was not statistically significant. Time from symptom onset to sample collection did not show a significant difference between AIS and ICH (Table 1).

**Table 1:** Patient characteristics according to stroke subtype.

	Intracerebral hemorrhage (n = 15)	Acute ischemic stroke (n = 48)	p-value
Age (years)	62 (53–71)	77 (66–83)	**0.02**
Sex			0.76
Female	5 (33.3%)	20 (41.7%)	
Male	10 (66.7%)	28 (58.3%)	
Diabetes mellitus	3 (%)	10 (%)	0.73
Serum creatinine (mg/dl)	1.02 ± 0.17	2.69 ± 1.53	0.16
Etiology	• Lobar 4 (26.7%) • Non-lobar (deep or infratentorial) 11 (73.3%)	• Large artery atherosclerosis 14 (29.2%) • Cardioembolism 23 (47.9%) • Small artery occlusion 3 (6.25%) • Other determined cause 1 (2.1%) • Undetermined cause 7 (14.6%)	
NIHSS			
At admission	15 (6–19)	11 (5–27)	0.4
At discharge	12 (1–20), n = 15	4 (2–12), n = 45	0.16
Time from symptom onset to sample collection (hours)*	26 (15–77), n = 12	40 (25–55), n = 37	0.29
Recanalization therapy	Not applicable	• Intravenous thrombolysis 20 (%) • Mechanical thrombectomy 24 (%)	
mRS			
Pre-stroke	0 (0–2)	1 (0–2)	0.4
At discharge	5 (4–5)	4 (2–5)	0.1
At 3 months	4 (3–6), n = 13	4 (2–5), n = 43	0.5
In-hospital mortality	3 (20%)	4 (8.3%)	0.3

Statistically significant p-values are depicted in a bold font.

*NIHSS* National Institutes of Health Stroke Scale.

*If precise onset-time was available.

Median serum levels were lower in the ICH patients than in the AIS group for NfL: Δ = 10.31 pg/ml, p = 0.24; UCH-L1: Δ = 3.95 pg/ml, p = 0.56 and t-tau: Δ = 0.12 pg/ml, p = 0.5, while GFAP showed an increased median level in ICH compared to AIS patients (Δ = 1803 pg/ml, p = 0.45). Urine median levels were approximately the same for all biomarkers across both groups. However, all the differences mentioned were not statistically significant (supplemental Fig. S1). The corresponding comparisons using the biomarker-creatinine ratios (biomarker-CRs) can be found in the supplemental material (supplemental Fig. S2).

### Differences in mRS at discharge and after 3 months

**Fig. 2:**
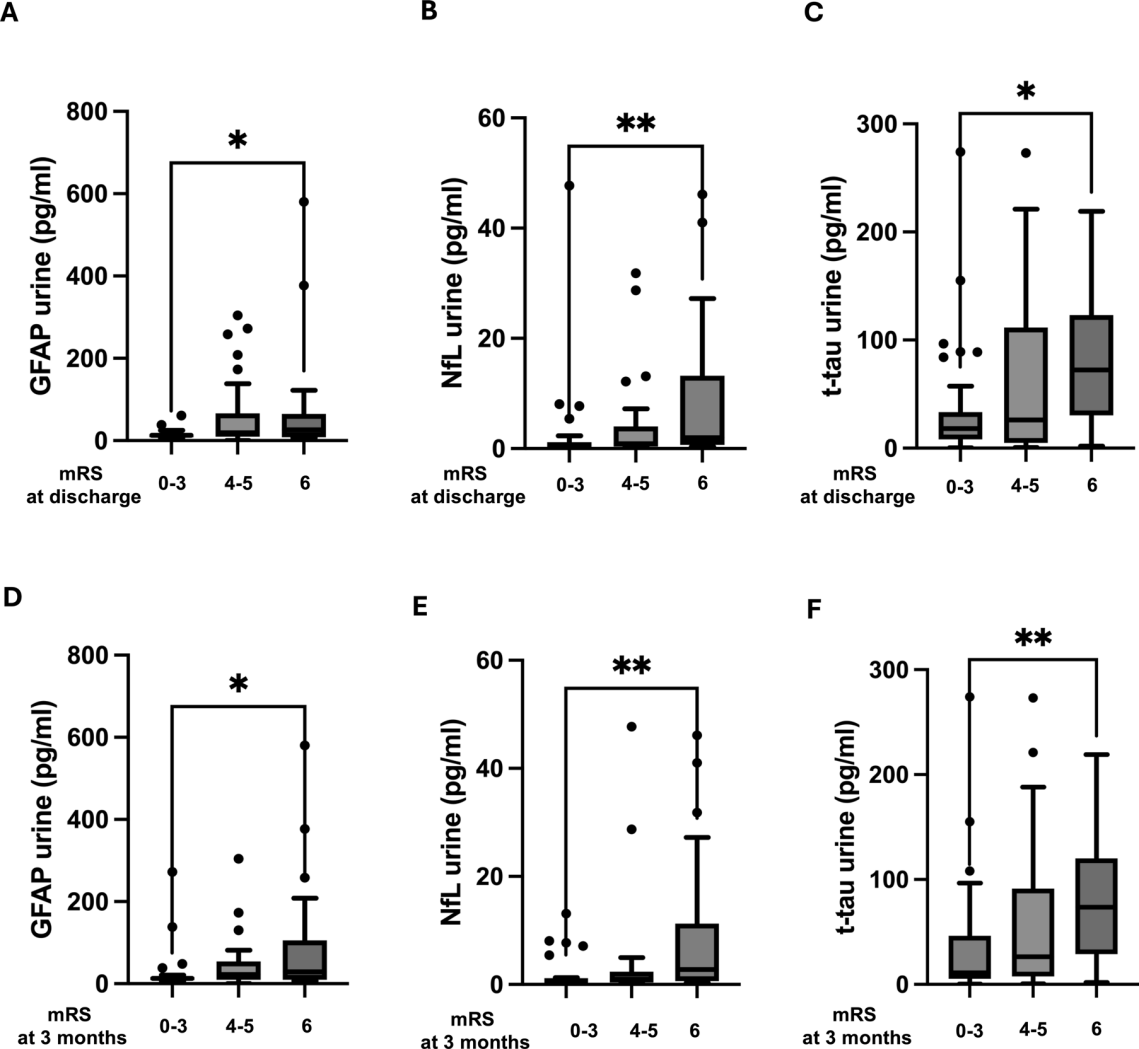
(A–C) GFAP, NfL and t-tau urine concentration according to mRS category at discharge. (D–F) GFAP, NfL and t-tau urine concentration according to mRS category at 3 months. Tukey box-and-whisker-plot.

We stratified the combined patient group according to their functional status at discharge and after 3 months by using the mRS categories at least fair outcome (mRS 0–3), poor to very poor outcome (mRS 4–5) and dead (mRS 6)^16^. We found a steady increase in the urine concentrations of GFAP, NfL and t-tau when stratified to the respective mRS categories with significant differences between patients with at least fair outcome and dead patients for NfL, t-tau and GFAP (GFAP: Δ 13.45, p = 0.02; Δ 15.95, p = 0.02; NfL: Δ 22.7, p = 0.009; Δ 20.4, p = 0.004; t-tau: Δ 20.2, p = 0.03, Δ 18.8, p = 0.01 at discharge and after 3 months, respectively; Fig. 2) and a reversed pattern for UCH-L1 with higher levels in the patients with at least fair outcome compared to patients with poor to very poor outcome and dead patients for both time points (at discharge and after 3 months). A detailed description of the single biomarker levels can be found in the supplemental material.

### Correlation and regression analysis of the functional outcome

A multivariate ordinal regression model was used to predict the mRS at discharge and at 3 months. In an initial Spearman correlation and univariate ordinal regression analysis to determine suitable covariates, serum and urine levels of GFAP, NfL, and t-tau were significantly associated with the mRS at discharge and at 3 months, whereas the NfL-CR was associated only with the mRS at discharge. A detailed summary is provided in Table 2, and correlations with functional outcomes for the individual patient groups (ICH and AIS) are presented in the supplemental material (Supplemental Table S1).

**Table 2:** Nonparametric Spearman correlations and univariate ordinal regression analysis for urine and serum biomarkers and biomarker-creatinine ratios, all patients together (intracerebral hemorrhage and acute ischemic stroke).

	n	Spearman correlation		Univariate ordinal regression	
		rho	p-value	Wald-Chi^2^	p-value
mRS discharge					
** GFAP urine**	**61**	0.23	0.07	**7.3**	**0.007**
** GFAP serum**	**61**	**0.27**	**0.03**	3.2	0.08
** NfL urine**	**60**	**0.33**	**0.01**	**4.1**	**0.04**
** NfL serum**	**61**	**0.34**	**0.01**	**5.2**	**0.02**
UCH-L1 urine	61	− 0.16	0.21	0.7	0.4
UCH-L1 serum	60	0.23	0.08	0.2	0.7
** t-tau urine**	**61**	**0.26**	**0.04**	**4.4**	**0.04**
** t-tau serum**	**61**	**0.27**	**0.04**	1.8	0.18
GFAP-crea ratio	50	0.01	0.94	3.8	0.05
** NfL-crea ratio**	**49**	0.18	0.22	**4.1**	**0.04**
UCH-L1-crea ratio	50	− 0.21	0.15	0.6	0.44
t-tau-crea ratio	50	0.25	0.08	2.9	0.09

mRS 3 months					
** GFAP urine**	54	0.25	0.07	**5.3**	**0.02**
** GFAP serum**	**54**	**0.30**	**0.03**	2.9	0.09
** NfL urine**	**53**	**0.43**	**0.002**	**6.4**	**0.01**
** NfL serum**	**54**	**0.4**	**0.003**	**8.2**	**0.004**
UCH-L1 urine	54	-0.11	0.44	0.5	0.49
UCH-L1 serum	53	0.23	0.1	1.8	0.18
** t-tau urine**	**54**	**0.27**	**0.04**	3.3	0.07
** t-tau serum**	**54**	**0.32**	**0.02**	1.7	0.2
GFAP-crea ratio	44	0.03	0.85	2.7	0.1
NfL-crea ratio	43	0.17	0.27	3.7	0.06
UCH-L1-crea ratio	44	-0.9	0.57	2.5	0.1
t-tau-crea ratio	44	0.25	0.1	0.9	0.4

The significant variables were entered into multivariable ordinal regression models with the mRS at discharge or at 3 months as the dependent variable, adjusted for confounders (NIHSS at admission, age at onset, and serum creatinine concentration). For the mRS at discharge, urinary GFAP was the most predictive covariate (Exp(B) [95% CI]: 1.005 [1.00–1.01]); however, it did not remain significant after correction for multiple testing (p = 0.049, q = 0.07). Urinary GFAP also showed a higher χ^2^ compared to serum GFAP (χ^2^ urine: 4.9, p = 0.027; χ^2^ serum: 1.08, p = 0.30). Serum and urinary NfL as well as urinary t-tau were not significantly associated with the mRS at discharge. For the mRS at 3 months, serum NfL was the only significant covariate (Exp(B) [95% CI]: 1.02 [1.00–1.04]; p = 0.02, q = 0.04) (Table 3). Across all multivariable ordinal regression models, NIHSS was the strongest predictor, showing consistently higher χ^2^ values and Exp(B) compared to any serum or urine biomarker. In a final regression model including both GFAP and NfL in serum and urine, urinary GFAP remained the most predictive biomarker for mRS at discharge, whereas serum NfL remained the most predictive for mRS at 3 months (Table 3).

**Table 3:** Multivariate ordinal regression models with mRS at discharge and at 3 months as dependent variables. Separate regression models were calculated for serum and urine biomarkers that were significant in the univariate regression analysis (Table 2). Each model was adjusted for NIHSS at admission, age at onset, and serum creatinine (I – VI). To improve readability, only the results for biomarker measurements and NIHSS at admission are reported. Furthermore, a combined multivariate regression model including serum and urine NfL and GFAP is presented (VII), likewise adjusted for the same confounders. The χ^2^ statistic indicates the likelihood ratio chi-square of each variable within the regression model, reflecting its contribution to the overall model fit. Odds ratios are expressed as Exp(B) [95%-confidence interval]. For each model, p-values were corrected for multiple testing using the False Discovery Rate (FDR) method and reported as q-values.

	Multivariate ordinal regression analysis									
	mRS discharge					mRS 3 months				
		n	χ^2^ [p-value]	Exp(B) [95%-CI]	p-value [q-value]		n	χ^2^ [p-value]	Exp(B) [95%-CI]	p-value [q- value]
**I**	**GFAP urine**	60	**4.9** **[0.027]**	**1.005** **[1.0–1.01]**	**0.049** [0.07]	**GFAP urine**	53	3.7 [0.06]	1.006 [1.0–1.01]	0.09 [0.1]
	**NIHSS**	60	**19.1** **[0.001]**	**1.16** **[1.08–1.24]**	** < 0.001** **[0.004]**	**NIHSS**	53	**13.1** **[0.001**]	**1.14** **[1.06–1.22]**	** < 0.001** **[0.004]**
**II**	**GFAP serum**	60	1.08 [0.3]	1.0 [1.0–1.0]	0.3 [0.4]	**GFAP serum**	53	1.6 [0.2]	1.0 [1.0–1.0]	0.2 [0.2]
	**NIHSS**	60	**26.0** **[0.001]**	**1.17** **[1.1–1.25]**	** < 0.001** **[0.004]**	**NIHSS**	53	**14.3** **[0.001]**	**1.13** **[1.06–1.21]**	** < 0.001** **[0.004]**
**III**	**NfL urine**	59	1.3 [0.26]	1.03 [0.98–1.09]	0.3 [0.4]	**NfL Urine**	52	**3.2** **[0.02]**	1.06 [0.99–1.14]	0.08 [0.1]
	**NIHSS**	59	**21.10.001]**	**1.16** **[1.09–1.25]**	** < 0.001** **[0.004]**	**NIHSS**	52	**15.0** **[0.001]**	**1.16** **[1.07–1.25]**	** < 0.001** **[0.004]**
**IV**	**NfL serum**	60	2.5 [0.11]	1.01 [1.0–1.03]	0.1 [0.2]	**NfL Serum**	53	**5.97** **[0.015]**	**1.02** **[1.0–1.04]**	**0.02** **[0.04]**
	**NIHSS**	60	**28.0** **[0.001]**	**1.18** **[1.1–1.26]**	** < 0.001** **[0.004]**	**NIHSS**	53	**15.7** **[0.001]**	**1.14 [1.07–1.22]**	** < 0.001** **[0.004]**
**V**	**NfL-CR**	48	1.5 [0.22]	–	0.2 [0.3]					
	**NIHSS**	48	**13.6** **[0.001]**	**1.14** **[1.06–1.22]**	** < 0.001** **[0.004]**					
**VI**	**t-tau urine**	60	1.4 [0.24]	1.0 [1.0–1.01]	0.2 [0.3]					
	**NIHSS**	60	**20.6** **[0.001]**	**1.16** **[1.08–1.24]**	** < 0.001** **[0.004]**					
**VII**	**GFAP serum**	54	1.9 [0.16]	1.0 [1.0–1.0]	0.2 [0.2]	**GFAP Serum**	47	3.2 [0.075]	1.0 [1.0–1.0]	0.08 [0.1]
	**GFAP urine**	**54**	**5.1** **[0.02]**	**1.01** **[1.01–1.02]**	**0.03** [0.1]	**GFAP Urine**	47	**4.2** **[0.04]**	1.01 [1.0–1.02]	0.06 [0.1]
	**NfL serum**	54	2.7 [0.1]	1.02 [1.0–1.05]	0.1 [0.2]	**NfL Serum**	**47**	**5.1** **[0.02]**	**1.04** **[1.0–1.07]**	**0.03** [0.1]
	**NfL urine**	54	0.05 [0.83]	0.99 [0.93–1.06]	0.8 [0.8]	**NfL Urine**	47	0.02 [0.88]	0.99 [0.9–1.09]	0.9 [0.9]
	**NIHSS**	**54**	**19.3** **[0.001]**	**1.17** **[1.08–1.26]**	** < 0.001** **[0.007]**	**NIHSS**	**47**	**12.0** **[0.001]**	**1.15** **[1.06–1.25]**	**0.001** **[0.007]**

### Correlation and regression analysis of infarct size and hematoma volume

**Fig. 3:**
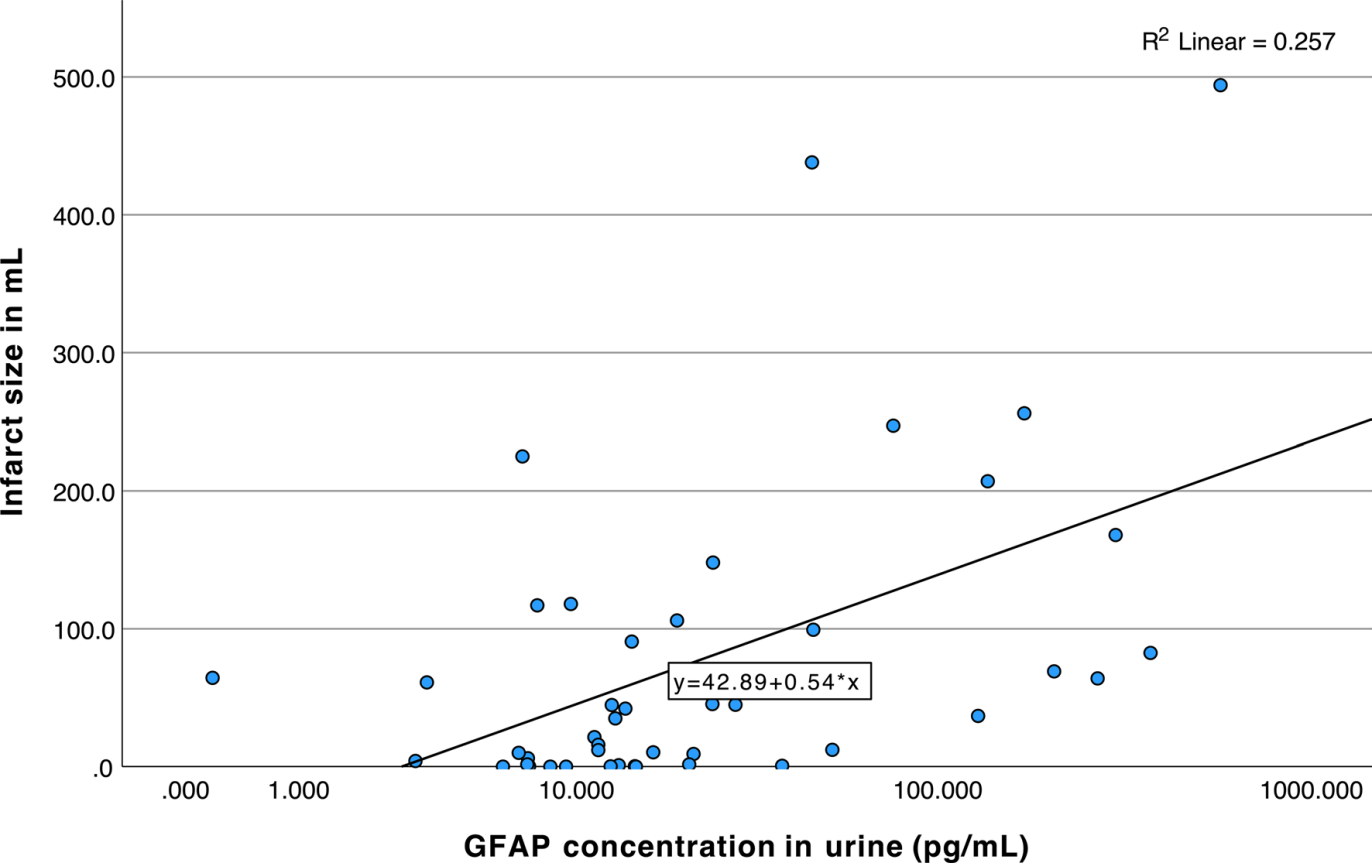
Scatterplot between the GFAP concentration (pg/ml) in urine and the infarct size in mL after acute ischemic stroke.

In the AIS patient group, urine and serum GFAP, serum UCH-L1 and serum t-tau correlated best with the ischemic lesion size (in ml as determined by volumetry, Table 4). In a subsequent linear regression model with correction to age at onset, NIHSS at admission and creatinine in serum, only GFAP in urine remained significant with an Exp(B) [95%-CI] of 1.56 [1.19–2.05] (p-value: 0.001, q-value: 0.007) (Table 5). The dependency between the GFAP concentration in urine and the infarct size is visualized using as a scatterplot (Fig. 3).

**Table 4:** Nonparametric Spearman correlations for imaging outcome (ischemic lesion size in milliliters as determined by volumetry) with urine and serum biomarkers and the biomarker-creatinine ratios, individually for patients with acute ischemic stroke or intracerebral hemorrhage).

Infarct size (mL)				
	n	rho	p-value	q-value
**GFAP urine**	**42**	**0.42**	**0.006**	**0.018**
GFAP serum	**45**	**0.6**	** < 0.0001**	**0.001**
NfL urine	41	0.13	0.42	0.5
NfL serum	45	0.22	0.15	0.24
UCH-L1 urine	42	0.22	0.16	0.24
**UCH-L1 serum**	**39**	**0.53**	**0.0005**	**0.003**
t-tau urine	42	0.07	0.68	0.68
**t-tau serum**	**45**	**0.43**	**0.004**	**0.016**
**GFAP-crea ratio**	**33**	**0.39**	**0.02**	**0.04**
NfL-crea ratio	34	0.16	0.39	0.5
**UCH-L1-crea ratio**	**34**	**0.41**	**0.02**	**0.04**
t-tau-crea ratio	34	0.11	0.53	0.57

Intracerebral hematoma volume (mL)				
	n	rho	p-value	q-value
GFAP urine	14	0.13	0.65	0.97
**GFAP serum**	**14**	**0.75**	**0.003**	**0.02**
NfL urine	14	0.04	0.9	0.97
NfL serum	14	0.01	0.97	0.97
UCH-L1 urine	14	− 0.06	0.84	0.97
**UCH-L1 serum**	**14**	**0.61**	**0.02**	0.09
t-tau urine	14	− 0.1	0.75	0.97
t-tau serum	14	0.41	0.15	0.39
GFAP-crea ratio	18	0.031	0.90	0.90
NfL-crea ratio	18	0.17	0.51	0.77
UCH-L1-crea ratio	18	− 0.16	0.53	0.77
t-tau-crea ratio	18	0.14	0.58	0.77

P-values were corrected for multiple testing using the False Discovery Rate (FDR) method and reported as q-values.

**Table 5:** Multivariate linear regression analysis of the infarct size (I) and hematoma volume (II) in milliliters as dependent variable (after ischemic stroke or intracerebrale hemorrhage, respectively) and multiple independent variables as predictors, which were identified using an initial spearman correlation. For confounding correction, age at onset, creatinine in serum and NIHSS at admission were included; the results for biomarker measurements and NIHSS at admission are reported. The χ^2^ statistic indicates the likelihood ratio chi-square of each variable within the regression model, reflecting its contribution to the overall model fit. Odds ratios are expressed as Exp(B) [95%-confidence interval]. P-values were corrected for multiple testing using the False Discovery Rate (FDR) method and reported as q-values.

	Infarct size (mL)					
		n	χ^2^ [p-value]	Exp(B) [95%-CI]	p-value	q-value
**I**	** GFAP urine**	40	**9.2 [0.002]**	**1.56 [1.19–2.05]**	**0.001**	**0.007**
	GFAP serum	40	0.3 [0.6]	1.00 [0.99–1.01]	0.58	0.67
	UCH-L1 serum	40	1.7 [0.2]	0.64 [0.33–1.25]	0.19	0.35
	t-tau serum	40	1.6 [0.2]	–	0.2	0.35
	NIHSS	40	2.1 [0.2]	22.2 [0.34–1443.29]	0.15	0.35

	Intracerebral hematoma volume (mL)					
		n	χ^2^ [p-value]	Exp(B) [95%-CI]	p-value	q-value
**II**	GFAP serum	14	0.2 [0.7]	1.0 [1.0–1.01]	0.65	0.86
	UCH-L1 serum	14	0.04 [0.8]	0.81 [1.11–6.21]	0.84	0.86
	** NIHSS**	**14**	**6.4 [0.01]**	**7.0 [1.84–26.68]**	**0.004**	**0.02**

In the ICH group, a Spearman correlation revealed a significant, positive correlation between ICH volume and GFAP in serum (Spearman ρ: 0.75, q-value: 0.02) (Table 4). However, after inclusion into a multivariate linear regression model with NIHSS at admission, age at onset and creatinine in serum, only NIHSS at admission remained the most strongest and independent predictors of the ICH volume (Exp(B) 7.0 [1.84–26.68], q-value: 0.02) (Table 5).

### Prediction of mortality

**Fig. 4:**
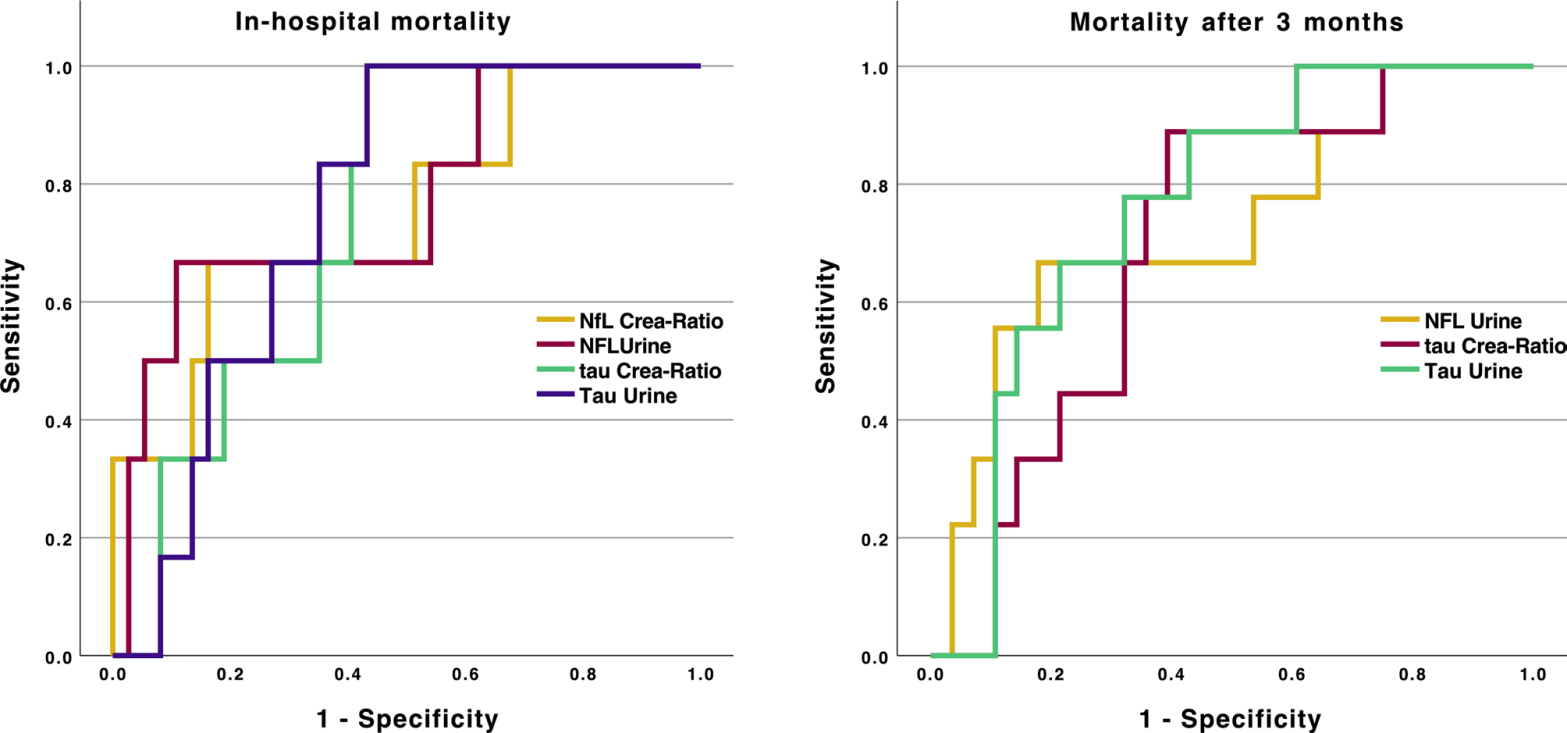
ROC curve analysis of significant urine biomarkers for the prediction of in-hospital mortality (left) and mortality at 3 months (right).

In the ROC curve analysis of the individual urine and serum biomarker levels for the prediction of in-hospital mortality (Table 6), we identified NfL in urine as well as tau in serum and urine as significant markers. For the prediction of mortality after 3 months, GFAP, NfL and t-tau in urine were significant predictors. In a combined ROC analysis with all significant biomarkers, NfL in urine as well as t-tau in urine and serum remained significant with an AUC of 0.77 [0.6–0.94] (p = 0.003) for NfL in urine, 0.79 [0.6–0.94] (p = 0.042) for t-tau in urine and 0.70 [0.51–0.9] (p = 0.042) for t-tau in serum. For the prediction of mortality after 3 months, only NfL in urine remained significant with an AUC of 0.74 [0.58–0.90] (p = 0.009) (Table 6). The ROC curves are visualized in Fig. 4.

**Table 6:** Receiver operating characteristic (ROC) curve analysis for the prediction of in-hospital or 3-month mortality, depending on the biomarkers in urine or serum.

In-hospital mortality			Combined				
	AUC	p-value	AUC [95%-CI]	p-value	Cutoff (pg/ml)	Sn %	Sp %
GFAP serum	0.63	0.18	–				
GFAP urine	0.68	0.08	–				
NfL serum	0.64	0.24	–				
NfL urine	0.72	**0.018**	**0.77 [0.6–0.94]**	**0.003**	**1.8**	**75.0**	**71.2**
UCH-L1 serum	0.61	0.22	–				
UCH-L1 urine	0.43	0.51	–				
t-tau serum	0.71	**0.015**	**0.70 [0.51–0.9]**	**0.042**	**0.52**	**75.0**	**0.68**
t-tau urine	0.71	**0.017**	**0.79 [0.6–0.94]**	**0.003**	**29.1**	**100.0**	**57.4**
GFAP-crea ratio	0.68	0.15	–				
NfL-crea ratio	0.59	0.48	–				
UCH-L1-crea ratio	0.47	0.82	–				
t-tau-crea ratio	0.65	0.17	–				

Mortality after 3 months			AUC [95%-CI]	p-value	Cutoff (pg/ml)	Sn %	Sp %
GFAP serum	0.60	0.22	–				
GFAP urine	0.69	**0.038**	0.68 [0.50–0.86]	0.051	26.6	69.2	72.5
NfL serum	0.58	0.42	–				
NfL urine	0.74	**0.003**	**0.74 [0.58–0.90]**	**0.009**	**1.76**	**69.2**	**75.0**
UCH-L1 serum	0.53	0.73	–				
UCH-L1 urine	0.49	0.91	–				
t-tau serum	0.60	0.24	–				
t-tau urine	0.68	**0.035**	0.67 [0.50–0.84]	0.051	97.95	53.8	82.5
GFAP-crea ratio	0.51	0.96	–				
NfL-crea ratio	0.56	0.63	–				
UCH-L1-crea ratio	0.50	0.98	–				
t-tau-crea ratio	0.65	0.12	**–**				

In a first step, the respective biomarkers were evaluated alone with a subsequent analysis of all significant biomarkers in one ROC analysis. P-values of the combined model were corrected for multiple testing.

*AUC* area under the curve, *Sn* sensitivity, *Sp* specificity, *crea* creatinine.

## Discussion

Our study offers insights into the utility of urine-based biomarkers for predicting outcomes following acute cerebral damage. Specifically, our findings suggest that NfL, t-tau and GFAP, when detected in urine, could serve as valuable indicators of neurological and tissue outcomes. To our knowledge, this is the first study to analyse the functional outcome after AIS or ICH based on neuronal or glial biomarkers in urine.

Among the urinary biomarkers, absolute GFAP concentration showed the strongest predictive value for mRS at discharge and, to a lesser extent, at 3 months following AIS or ICH. Its performance was superior to serum GFAP within the same time frame, as indicated by higher χ^2^ values for model fit (Table 3). In contrast, although NfL correlated significantly with mRS at both time points, these associations did not remain significant after adjustment for confounders and were inferior to urinary GFAP for predicting in-hospital- and 3-month functional outcomes. Regarding serum biomarkers, only serum NfL remained a significant predictor of 3-month mRS, where it outperformed urinary NfL concentrations.

When models were adjusted for established risk factors, NIHSS at admission remained the strongest predictor, surpassing all biomarker measurements. Nevertheless, urinary GFAP contributed additional and independent predictive value for in-hospital functional outcome, while serum NfL independently predicted 3-month outcome (Table 3). For in-hospital mortality, urinary NfL as well as serum and urinary t-tau were significant predictors, whereas only urinary NfL remained significant for 3-month mortality (Table 6).

Surprisingly, the serum biomarker concentrations only showed a weak correlation with the in-hospital mortality and mortality after 3-months, which especially after inclusion into a multivariate regression model remained in-significant for all biomarkers and did not add additional value for the prediction of functional outcomes. A possible explanation might be the time of sample acquisition, which allowed later times compared to similar studies^8,17,18^. Furthermore, using the biomarker-creatinine ratio (which is supposed to be a more valid and accurate method as the values are corrected for urine dilution^19^), did not provide additional predictive value compared to the absolute urine biomarker concentrations. A possible explanation is that absolute biomarker concentrations in urine may increase in the presence of glomerular impairment or highly concentrated urine (e.g., due to dehydration or renal failure), both of which are also associated with poorer functional outcomes after ischemic stroke^20^. In such cases, elevated absolute urine biomarker levels might reflect not only the extent of brain injury but also acute kidney failure. Adjusting for these factors through a biomarker-to-creatinine ratio may therefore diminish the predictive value of absolute concentrations. To partially account for this, we included serum creatinine levels in the multivariate regression model; however, the exact contribution of acute renal failure was not fully evaluated in our study.

In patients with AIS, GFAP in urine showed the best correlation with the ischemic lesion size and is in line with previous data about GFAP in serum as a marker of astroglial injury^9^ to assess tissue damage in brain injuries^4,21^. In this context, the detectability in urine^11^ represents an intriguing finding, which adds to the existing literature about GFAP following astrocytic injury^22,23^ and could assist clinicians in assessing tissue-specific outcomes following AIS.

We did not find significant differences of the absolute urine biomarker concentrations between the respective underlying etiologies, which strengthens our approach of a composite group of AIS and ICH for the statistical outcome analysis. On the downside, a distinction between ICH and AIS could not be made based solely on the urine biomarker determination alone within the chosen timeframe of sample collection. In particular, a differentiation between ICH and AIS is especially possible within the first 6 h after symptom onset with a subsequent alignment of the serum levels between the etiologies^24^. In our study, only a minority of our blood and serum samples were drawn within 24 h after symptom onset, none within 6 h. In this context, the estimated serum peak, converging between AIS and ICH in the chosen timeframe, was selected to improve prognostic assessment, accepting a lower threshold to distinguish between the two entities. It is worth noting, that, as expected, there was a strong correlation between GFAP in serum and ICH volume, but contrary to the correlation in AIS this could not be identified in urine. Besides the lower number of cases, a different release of GFAP into blood and subsequently into urine could be assumed as possible explanations for this finding.

In clinical practice, the assessment of stroke severity und thus outcome prediction can be challenging in the subacute phase following an acute stroke as concomitant extracerebral diseases and systemic (such as infections) or cerebral complications (such as epileptic seizures) may contribute to a worse outcome. Urine biomarkers offer advantages in terms of accessibility, safety, and the ability to conduct repeated assessments without invasive procedures. This could be particularly beneficial in populations where blood draws are challenging, such as pediatric or elderly patients as well as regions with poor medical infrastructure. Despite the sensitivity and specificity of urine biomarkers in this study were moderate, they were overall superior to the respective predictive value of serum in our cohort. However, prior publications, showed sligthly higher AUC values for predicting short term mortality and functional outcomes (ranging from 0.78 to 0.88 for GFAP, 0.681–0.812 for NfL, 0.81–0.76 for t-tau and 0.86–0.89 for UCH-L1)^17,25–29^. In our study, the AUC values for predicting mortality (ranging from 0.68 to 0.74 for GFAP, NfL, and t-tau) suggest that urine biomarkers can provide valuable information, but they may not yet be a standalone diagnostic tool. Given the moderate sensitivity and specificity observed, urine biomarkers may be best used in conjunction with clinical assessments and imaging, rather than as a replacement for them. With ongoing advances in imaging techniques, many new imaging biomarkers have gained attention in recent years. These have been shown to correlate with clinical and tissue outcomes and can assist in determining the penumbra, thereby influencing treatment decisions^30,31^. The combination of imaging and classical serum biomarkers is already used in other areas, and will be increasingly applied in in stroke, yielding enhanced diagnostic and prognostic accuracy^32–34^.

The variability in biomarker levels among patients and across different types of brain injury highlights the complexity of interpreting urine biomarkers. Furthermore, pre-existing neurological diseases, acute infections, kidney function, and concomitant medication can all influence biomarker levels in acute stroke patients. Future studies should thus also explore the potential confounding factors that may affect the excretion of these biomarkers in urine, such as renal function, hydration status, and age. A more comprehensive understanding of the dynamics of these biomarkers in urine, including their stability, half-life, and elimination pathways, will be essential for optimizing their use in clinical practice. Additionally, longitudinal studies tracking urine biomarker levels over time could provide insights into the temporal progression of cerebral damage and recovery. A more detailed assessment of biomarker kinetics would therefore ideally require measurements at several time points, such as 24, 48, 72, and 96 h this would help to assess whether a sustained increase might correlate with clinical severity.

In addition, the data was collected within 96 h during hospitalization and therefore were performed outside the normal time window for treatment decisions. The benefit of urine biomarker measurements in the hyperacute phase to direct therapeutic approaches therefore remains unclear and requires further investigation in the future. In this case, it would be of particular interest to what extent urine biomarkers can be used to differentiate between AIS and ICH, or whether there is a correlation with the early extent of infarction or ICH.

A weakness of this study is the relatively small group size, especially in the evaluations for lesion size (tissue outcome), as the ICH group was generally much smaller, resulting in a limited statistical power. However, given that we found significant and plausible effects for many of the parameters of interest, this pilot study offers a foundation for larger studies.

Our study evaluated neuro-glial biomarkers in serum and urine within 96 h of symptom onset for predicting in-hospital and 3-month functional outcomes in patients with AIS or ICH. Among the biomarkers, urinary GFAP showed the strongest predictive value for in-hospital functional outcome, which was higher than the respective serum concentrations and remained independently associated with outcome alongside NIHSS at admission. While urinary NfL showed a trend toward additional predictive value for 3-month outcome, this effect was stronger and statistically significant for serum NfL. For mortality prediction, urinary NfL was the only biomarker consistently associated with both in-hospital and 3-month outcomes. In conclusion, urinary GFAP and NfL may serve as potential non-invasive biomarkers for prognostic assessment in acute stroke, possibly providing additional information beyond established clinical risk factors.

## Supplementary Information

Supplementary Information.

## Data Availability

Data is provided within the manuscript or supplementary information files. Source data will be made available upon reasonable request to the corresponding author (Dr. Kohlhase, kohlhase@med.uni-frankfurt.de).
