# Supplementary material for: The use of brain-specific biomarkers in urine for prediction of neurological outcome and extent of tissue damage following stroke
Source: Sci Rep. 2025 Dec 3;15:43089. doi: 10.1038/s41598-025-28115-1 (PMC12675713; doi:10.1038/s41598-025-28115-1)
Supplement: Supplementary file 1 — Supplementary Information. [file 41598_2025_28115_MOESM1_ESM.docx]

**Supplemental**

**Absolute biomarker levels according to stroke subtype (ICH vs. AIS)**

**Figure S1:** Tukey box-and-whisker-plot **A-H** protein biomarker levels according to stroke subtype. ICH: n= 15, AIS: n= 48. Bar graphs indicating mean ± SEM. AIS= acute ischemic stroke, ICH= intracerebral hemorrhage.

**Biomarker-creatinine ratios according to stroke subtype (ICH vs. AIS)**

**FigureS2:** Tukey box-and-whisker-plot, A-D biomarker-creatinine ratios according to stroke-subtypes (ICH vs. AIS). ICH= intracerebral hemorrhage, AIS= acute ischemic stroke.

**Urine biomarker levels stratified to mRS groups**

The median urine biomarker concentration (Q1– Q3) of patients with at least fair outcome at discharge (mRS 0-3) was 12.7 (8.5–15.6) pg/ml for GFAP, 0.6 (0.3– 1.2) pg/ml for NfL, 72.2 (18.9– 148) pg/ml for UCH-L1, and 18 (8.1– 33.4) pg/ml for t-tau. The median urine biomarker concentration of the patients with poor to very poor outcome (mRS 4-5) was 18.8 (9.6– 66.5) pg/ml for GFAP, 0.9 (0.4– 4.1) pg/ml for NfL, 47.6 (22.9– 85.8) pg/ml for UCH-L1, and 26 (4.9– 111.5) pg/ml for t-tau. Finally, the median urine biomarker concentration of the patients with intrahospital death (mRS 6) was 26.2 (8.7– 65.2) pg/ml for GFAP, 2 (0.7– 13.2) pg/ml for NfL, 35.2 (10.9– 79.1) pg/ml for UCH-L1, and 72.3 (30.3– 123) pg/ml for t-tau. The median urine biomarker concentration of the patients with fair outcome after 3 months (mRS 0-3) was 13.1 (9.3– 16.5) pg/ml for GFAP, 0.6 (0.2– 1.2) pg/ml for NfL, 74.2 (32.9– 165) pg/ml for UCH-L1, and 11.1 (5.3– 46.3) pg/ml for t-tau. The median urine biomarker concentration of the patients with poor to very poor outcome (mRS 4-5) was 22.1 (9.4– 54.6) pg/ml for GFAP, 1.0 (0.4– 2.4) pg/ml for NfL, 39 (18– 39) pg/ml for UCH-L1, and 26.5 (7.6– 91.5) pg/ml for t-tau. Finally, the median urine biomarker concentration of the patients with death at three months (mRS 6) was 29.1 (9.7– 105.6) pg/ml for GFAP, 2.8 (0.6– 11.3) pg/ml for NfL, 39.9 (16– 102.1) pg/ml for UCH-L1, and 73.6 (29– 120) pg/ml for t-tau.

**Correlation to functional outcome for the individual patient groups**

In the AIS patient group, urine GFAP and NfL correlated best with functional outcome at discharge (measured by mRS), although this was no longer statistically significant after correction for multiple correlations. Urine and serum NfL correlated significantly with the mRS 3 months later. In the ICH group, after correction none of the serum and urine biomarkers correlated significantly with the functional outcome at discharge, however we identified serum UCH-L1 and t-tau significantly correlating with the functional outcome at 3 months (Table S1).

|  | n | rho | p value | Benjamini-Hochberg adjusted p value |
| --- | --- | --- | --- | --- |
| **Acute ischemic stroke (AIS)** | | | | |
| **mRS discharge** | | | | |
| **GFAP urine** | **44** | **0.3013** | **0.0469** | 0.1876 |
| GFAP serum | 45 | 0.2052 | 0.1762 | 0.2819 |
| **NfL urine** | **43** | **0.4078** | **0.0066** | 0.0528 |
| NfL serum | 45 | 0.2493 | 0.0986 | 0.1972 |
| UCH-L1 urine | 44 | -0.1086 | 0.4827 | 0.4827 |
| UCH-L1 serum | 44 | 0.1467 | 0.3419 | 0.2907 |
| t-tau urine | 44 | 0.2719 | 0.0742 | 0.1972 |
| t-tau serum | 45 | 0.1687 | 0.2679 | 0.3572 |
| **mRS 3 months** | | | | |
| GFAP urine | 39 | 0.3028 | 0.0609 | 0.1218 |
| GFAP serum | 40 | 0.2295 | 0.1542 | 0.2467 |
| **NfL urine** | **38** | **0.4857** | **0.0020** | **0.016** |
| **NfL serum** | **40** | **0.3179** | **0.0456** | 0.1218 |
| UCH-L1 urine | 39 | 0.0338 | 0.8383 | 0.8383 |
| UCH-L1 serum | 39 | 0.0849 | 0.6074 | 0.6942 |
| t-tau urine | 39 | 0.3039 | 0.0600 | 0.1218 |
| t-tau serum | 40 | 0.1987 | 0.2189 | 0.2919 |
| **Intracerebral hemorrhage (ICH)** | | | | |
| **mRS discharge** | | | | |
| GFAP urine | 14 | 0.1454 | 0.6178 | 0.7061 |
| GFAP serum | 14 | 0.3672 | 0.1956 | 0.3507 |
| NfL urine | 14 | 0.2862 | 0.3188 | 0.4251 |
| NfL serum | 14 | 0.5114 | 0.0640 | 0.256 |
| UCH-L1 urine | 14 | 0.0591 | 0.8418 | 0.8418 |
| UCH-L1 serum | 14 | 0.4406 | 0.1072 | 0.2859 |
| t-tau urine | 14 | 0.3498 | 0.2192 | 0.3507 |
| **t-tau serum** | **14** | **0.6011** | **0.0255** | 0.204 |
| **mRS 3 months** | | | | |
| GFAP urine | 12 | 0.3221 | 0.3042 | 0.3477 |
| **GFAP serum** | **12** | **0.6007** | **0.0428** | 0.1141 |
| NfL urine | 12 | 0.3972 | 0.2006 | 0.3209 |
| NfL serum | 12 | 0.4777 | 0.1179 | 0.2358 |
| UCH-L1 urine | 12 | -0.1861 | 0.5591 | 0.5591 |
| **UCH-L1 serum** | **12** | **0.7672** | **0.0053** | **0.0212** |
| t-tau urine | 12 | 0.3471 | 0.2670 | 0.3477 |
| **t-tau serum** | **12** | **0.8809** | **0.0003** | **0.0024** |

**Table S1** Nonparametric spearman correlations for functional and imaging outcome with urine and serum biomarkers, individually for ICH and AIS patient groups, healthy subjects excluded).

**Sensitivity Analysis excluding patients with serum UCH-L1 values below the limit of detection**

Speaman correlation to mRS at discharge: n= 55, rho 0.2, p=0.1. Correlation to mRS at 3 months: n=48, rho 0.2, 0.2
